# How Do Molecular Dynamics Data Complement Static Structural Data of GPCRs

**DOI:** 10.3390/ijms21165933

**Published:** 2020-08-18

**Authors:** Mariona Torrens-Fontanals, Tomasz Maciej Stepniewski, David Aranda-García, Adrián Morales-Pastor, Brian Medel-Lacruz, Jana Selent

**Affiliations:** 1 Research Programme on Biomedical Informatics (GRIB), Hospital del Mar Medical Research Institute (IMIM)—Department of Experimental and Health Sciences, Pompeu Fabra University (UPF), 08003 Barcelona, Spain; mariona.torrens@upf.edu (M.T.-F.); tm.stepniewski@gmail.com (T.M.S.); darandagar@gmail.com (D.A.-G.); drnmoralespastor@gmail.com (A.M.-P.); brianmedelmo@gmail.com (B.M.-L.); 2 InterAx Biotech AG, PARK innovAARE, 5234 Villigen, Switzerland; 3 Faculty of Chemistry, Biological and Chemical Research Centre, University of Warsaw, 02-093 Warsaw, Poland

**Keywords:** GPCRs, molecular dynamics, ligand binding, receptor (in)activation, receptor signaling, drug discovery

## Abstract

G protein-coupled receptors (GPCRs) are implicated in nearly every physiological process in the human body and therefore represent an important drug targeting class. Advances in X-ray crystallography and cryo-electron microscopy (cryo-EM) have provided multiple static structures of GPCRs in complex with various signaling partners. However, GPCR functionality is largely determined by their flexibility and ability to transition between distinct structural conformations. Due to this dynamic nature, a static snapshot does not fully explain the complexity of GPCR signal transduction. Molecular dynamics (MD) simulations offer the opportunity to simulate the structural motions of biological processes at atomic resolution. Thus, this technique can incorporate the missing information on protein flexibility into experimentally solved structures. Here, we review the contribution of MD simulations to complement static structural data and to improve our understanding of GPCR physiology and pharmacology, as well as the challenges that still need to be overcome to reach the full potential of this technique.

## 1. Introduction

G protein-coupled receptors (GPCRs) are a large and versatile family of transmembrane proteins, encompassing over 800 identified members. These proteins act as receptors for a wide variety of extracellular stimuli including light, changes of pressure, and chemical ligands, odorants, neurotransmitters, chemokines, and metabolites among others, transducing their information into intracellular signaling cascades. Due to their participation in a wide range of pathways and physiological processes, as well as their druggability, GPCRs have become a drug target of major importance in the pharmaceutical industry [1].

As a consequence of their relevance for drug discovery, deciphering the molecular basis of GPCR signaling has become a major research focus. The signaling outcome of GPCRs is determined by their three-dimensional conformation, which is variable and depends on multiple factors, such as the binding of orthosteric and allosteric ligands, the lipidic environment, and post-translational modifications. Understanding how all of these factors contribute to a specific structure, and in turn, a specific signaling response, would not only expand our knowledge of GPCR biology but also provide structural blueprints for the design of novel and better therapeutics. To address this ambitious goal, numerous endeavors have been undertaken to characterize the three-dimensional structure of GPCRs and its changes over time.

**Figure 1:**
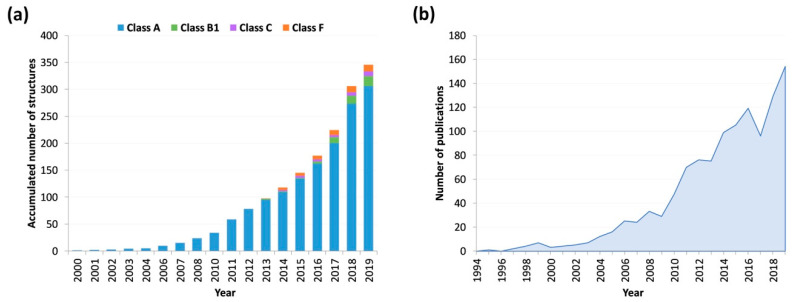
(**a**) Number of G protein-coupled receptors (GPCRs) structures available in GPCRdb [4,5] over time. (**b**) Number of publications per year indexed at Thomson Reuters’ Web of Science that contain the topics “molecular dynamics” and (“GPCR” or “GPCRs”). The exponential growth of successful GPCR research based on molecular dynamics (MD) simulations is evidenced by the rapid upsurge in the number of publications per year related to this subject.

Important advances in protein engineering, X-ray crystallography, and cryo-electron microscopy (cryo-EM) during the past decade have led to an exponential growth in the number of known GPCR structures. Since then, the number of available structures has continued increasing (Figure 1a). This large data set has been crucial for advancing our understanding of GPCR function. Moreover, it enabled the application of structure-based drug design approaches, which aid the discovery of novel drug candidates with improved pharmacological profiles [1,2,3].

Despite their enormous utility, high-resolution structures describe proteins mainly as rigid entities, whereas information about their intrinsic flexibility and conformational plasticity cannot be appreciated. With the goal to incorporate atomic-level dynamic information to static systems, molecular dynamics (MD) simulations were introduced several decades ago. The first MD simulation of a biomolecule was 9.2 ps-long and consisted of the bovine pancreatic trypsin inhibitor (~500 atoms) in vacuum [6]. In the case of GPCRs, the first MD simulation was obtained in 1991, before the first GPCR crystal structure was resolved [7]. It corresponded to an 80 ps-long trajectory of a rat dopamine D_2_ receptor, modeled from its sequence with molecular mechanics. Ever since, MD simulations have greatly improved their performance, allowing the simulation of larger systems for longer timescales. A major determinant of these advances has been the development of algorithms optimized for graphical processor units (GPUs), a technology first designed to improve video game performance [8,9]. GPU exploitation was a major breakthrough for the field, enabling researchers to perform on commodity hardware calculations that were previously only possible with the use of supercomputing clusters. Along with these technological advances, the expansion of free and user-friendly software for the input preparation (e.g., CHARMM-GUI [10], HomolWat [11]) and analysis (e.g., MDAnalysis [12,13]) of MD simulations has greatly contributed to the broad application of this technique.

Owing to the aforementioned technical developments, MD simulations currently provide a combination of temporal and structural resolution greater than what is usually achievable by experimental methods [14]. As a result, MD simulations are widely used for the study of GPCRs, as reflected by the continuous increase in publications per year on this topic (Figure 1b). Moreover, most publications on crystallography now supplement their studies with MD to refine the obtained structure. Here, we review recent developments in the study of GPCR functionality using MD simulations to complement static structural data. We discuss the role of receptor dynamics in several functional processes, outline the applicability of MD simulations for drug discovery, and describe the basis of this technique. We also examine the main challenges that still need to be overcome to reach its full potential. Finally, we discuss the future of the field. 

## 2. Complementing Static Data

Three-dimensional structures derived from experiments via X-ray crystallography or cryo-EM provide high-resolution information about specific conformational states of GPCRs. However, we need to be aware that these structures represent low energetic conformational states that are obtained under experimental conditions that often deviate from native-like conditions. In this scenario, MD simulations are a useful tool to drive these structures to conformational states that are linked to a more native-like environment. Moreover, MD simulations incorporate the missing information on structural motions, yielding insights that can be critical to the understanding of GPCR physiology and pharmacology [14]. In this respect, MD simulations have proven useful to complement static data and expand our knowledge of processes such as binding of small molecules or drugs to orthosteric or allosteric receptor sites. We can also determine how a biomolecular system will respond to perturbations such as mutations, post-translational modifications, and the composition of the cell membrane [15,16]. In addition, we can study the conformational rearrangements that occur during receptor (in)activation, determine metastable receptor states along the transition pathways or explore the interaction with intracellular coupling partners [17,18]. Even processes such as receptor dimerization/oligomerization, which has been implicated in fine-tuning GPCR signaling, can be investigated using different MD techniques [19].

### 2.1. Molecular Mechanism of Receptor Activation

From a structural perspective, there are two mechanisms by which a molecule, so-called “agonist”, can mediate GPCR activation. On the one hand, an agonist can sample and stabilize a subset of receptor conformations known as “active states”, shifting the conformational equilibrium to an active receptor (conformational selection mechanism) [20]. On the other hand, the binding of the agonist can initiate small structural changes in the ligand binding site, which are propagated across the receptor through rearrangements of specific residues. These rearrangements lead to global structural changes towards conformational populations of active receptor states (induced fit mechanism) [21]. Most likely, both mechanisms contribute to a different extent to receptor activation depending on the ligand and receptor type [14,22]. Finally, receptors in an active state have a higher propensity to interact with intracellular partners. This leads to the initiation of signaling cascades which ultimately alter the metabolism of the cell [23].

Experimentally solved structures provide extensive information on the conformation of several active, inactive, and intermediate states [24,25]. Such structures have been an excellent starting point for numerous MD simulation-based studies that clarify the activation/inactivation mechanism. By this means, researchers have been able to probe the flexibility of GPCR-ligand complexes in the initial and final stages of activation and observe structural fundamentals on how ligands stabilize conformational states that are related to specific signaling outcomes [26,27]. Furthermore, extending such simulations it is possible to capture intermediate conformations that are adopted on the transition pathway.

Beyond this, pioneering simulations on the active conformation of the β-2 adrenergic receptor (β_2_AR) [28] revealed that the presence of an intracellular coupling partner is crucial to stabilize the receptor in an active state. Without it, the receptor can revert to a fully inactive state, despite the presence of an agonist. The study also highlighted multiple structural features related to activation, which are loosely coupled and do not necessarily occur sequentially. These results were later supported by NMR data [29]. After these findings, a simulation of unprecedented total length, obtained thanks to Google’s Exacycle cloud computing platform, allowed the generation of a complete structural statistical model of GPCR activation [30]. One of the highlights of this study was that GPCRs can follow multiple pathways towards obtaining an active conformation. 

On a more detailed level, MD simulation also permits the study of more subtle structural rearrangements related to activation. A notable example includes a comprehensive study carried out by Li et al. [31]. By simulating complexes of the A_2A_ receptor (A_2A_R) with multiple ligands, they were able to obtain a comprehensive view of the activation mechanism of this receptor. Importantly, they observed that the conserved residue W6.48 attained different conformational states in response to agonists. Moreover, by studying receptor-ligand contacts, they were able to identify groups of contacts that lead to a specific signaling response. These interactions promoted local structural changes that led to the increased mobility of the transmembrane helix (TM) 6. Importantly, these results were in line with crystallographic [24] and NMR data [32].

Furthermore, post-translational modifications have been described to be critical for the biological activity of GPCRs. In this respect, Oddi et al. report for instance that the biological activity in terms of the CB_1_ receptor is closely linked to palmitoylation of cysteine 415 in helix 8 [33]. MD simulation revealed that this modification stabilizes helix 8 and promotes the binding of cholesterol molecules in the vicinity, which likely facilitates the interaction with lipid rafts and caveolin 1. This goes along with the experimental finding that the C415A mutation impairs the receptor’s ability to functionally interact with lipid rafts as well as eliminates agonist-dependent internalization of the CB_1_ receptor. In addition, the same group shows that palmitoylation of cysteine 415 fine-tunes CB_1_ receptor interaction with the Gαi2 protein, which further highlights the relevance of post-translational modifications for receptor functionality [34]. 

As integral membrane proteins, GPCRs communicate with the lipid environment, which contributes to the regulation of GPCR function and dynamics. Membrane phospholipids have been found to allosterically modulate the activity [35,36,37,38] and oligomerization [19] of GPCRs, while membrane cholesterol can regulate its stability, ligand-binding properties and function [16,39,40,41]. Still, the precise nature of lipid implication in GPCR modulation is unclear. Such effects can either be attributed to changes in membrane biophysical properties (including thickness, curvature, and surface tension) [42,43], direct interactions [15,44,45,46], or both. In one of the first MD studies comparing the effects of different single species lipid bilayers on the dynamical behavior of a GPCR, Ng. et al. showed that the structural motions of the A_2A_R may depend on its phospholipid environment [47]. This could be explained by the physical adaptation of the A_2A_R to different membrane thicknesses or by molecular interactions of the lipid headgroups and the protein. Similarly, in a recent study Bruzzese et al. examined how much different membranes affect the activation process of the A_2A_R and the functional effect of their agonists [48]. Based on microsecond-long MD simulations, they revealed an effect of the phospholipid membrane in the intermediate or active receptor conformations observed, which can be attributed to phospholipid-mediated allosteric effects on the intracellular side of the receptor. In addition to identifying potential lipid interaction sites, MD simulations can provide estimates of the free energy of protein-lipid interactions, which permits to quantify their strength. To test the reliability of MD to study the energetics of protein−lipid interactions, Corey et al. compared different MD-based approaches in terms of ease of accuracy and computational cost [49]. They showed that such methods produce estimates of the strength and specificity of lipid-binding sites that are robust and reproducible. 

Finally, a relatively recent finding is that GPCRs can couple to diverse intracellular signaling partners, including different G proteins and β-arrestins. An interesting observation is that, in some cases, only a subset of pathways is engaged upon ligand binding, a phenomenon known as “signaling bias” [50,51]. The underlying molecular mechanism of signaling bias is still poorly understood and will be addressed in more detail in a later section (Section 2.2.2). 

### 2.2. Ligand Binding to GPCRs

**Figure 2:**
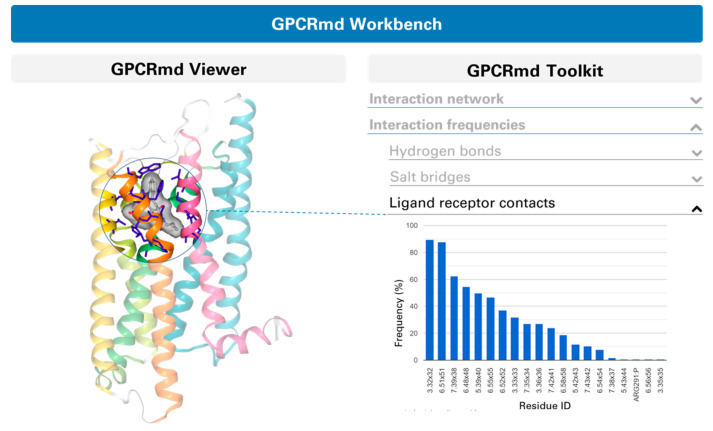
Schematic view of the ligand-protein interaction results that can be obtained with the GPCRmd server [53]. Specifically, the GPCRmd Workbench module of the server enables interactive visualization (GPCRmd Viewer) and analysis (GPCRmd Toolkit) for individual simulations, including ligand-protein interactions among others. Figure obtained from the GPCRmd server [53].

Typically, GPCRs are able to recognize and bind a variety of ligands that modulate the receptor functional outcome. Deciphering the complex process of receptor modulation, and how specific interactions in the ligand binding site are linked to the final functional outcome, has been a main goal of many scientific endeavors. Such information would help us better understand GPCR physiology and inform the design of molecules with a specific signaling profile [52]. A valuable resource of ligand binding dynamics is found in the recently established GPCRmd server [53], which provides intuitive visualization and analysis tools currently covering 70% of crystallized receptor subtypes (Figure 2).

#### 2.2.1. Classical Orthosteric Ligands

The use of static structures to understand ligand binding can lead to incomplete information, especially in receptors with high flexibility. This was highlighted by Ferruz et al. in a study where they compared the binding poses of several dopamine D_3_ receptor antagonists obtained with static docking and with MD simulations [54]. Using large-scale MD simulations and Markov state models (MSMs), they were able to overcome the limitations of docking in the determination of the ligand binding poses and revealed a cryptic binding pocket. Virtual screening protocols considering only static structures would miss compounds binding to this cryptic binding pocket. Thus, the characterization of the intrinsic flexibility of GPCRs is of great value for the identification or design of new ligands [55], as discussed also in Section 3.

Similarly, MD studies provide valuable information on the strength of ligand-receptor interactions in terms of contact frequencies that cannot be obtained by methods that do not account for the flexibility of the binding site. This information facilitates the identification of the key interactions that a ligand establishes in the binding pocket and which likely drives the signaling outcome. For example, a combination of molecular modeling and simulation was used to describe the binding characteristics of the natural agonist and its derivatives in the oxoeicosanoid receptor 1, providing new insights into how this receptor is modulated [56]. Moreover, MD simulation provided information on ligand stability and key interactions that allowed identifying selectivity features of 5-HT_2B_ fluorescent ligands that retain the agonistic functional behavior of the model ligand [57].

The interaction between a ligand and a GPCR, however, is not only determined by the events that happen once in the binding site. The ligand needs to pass through a series of intermediate states between the solution phase and the fully bound pose, known as the ligand binding pathway. Describing this pathway can lead to the identification of energetic barriers that affect the binding and unbinding rates. Ultimately, such rates play a pivotal role in drug efficacy, selectivity, and safety [58,59,60]. The details of the binding pathways are difficult to probe by experimental techniques, but MD simulations generate useful insights on this process [61]. Some highlights in the MD-based characterization of binding pathways include (S)-alprenolol binding to the β_2_AR [62], histamine to the histamine H_4_ receptor [63], adenosine to the A_2A_R [64], and clozapine and haloperidol to the dopamine D_2_ and D_3_ receptors [65]. Importantly, the case of the β_2_AR was the first unbiased MD simulation study capturing the full process of ligands spontaneously binding to a GPCR. Dror et al. were able to achieve final poses matching those determined crystallographically without the incorporation of any prior knowledge of the binding site. Results revealed not only the predominant pathway into the binding site, but also the two main energetic barriers that govern drug binding and unbinding kinetics. 

#### 2.2.2. Biased Agonists

Biased agonists are molecules of high interest, as they selectively target a specific signaling pathway in a cell while maintaining other signals in their physiological state. Biased signaling probes are valuable tools to interrogate the involvement of the pathway in physiological processes or in the development of disease symptoms. Furthermore, they are promising starting points for the development of safer drugs, as they potentially allow selective modulation of pathways associated with disease symptoms while not engaging counter-therapeutic pathways or those related to debilitating side-effects. 

Several studies demonstrated the usefulness of MD simulations to uncover distinct molecular events that are linked to a biased response [66]. Thus, Martí-Solano et al. characterized the dynamic receptor interaction fingerprint of biased agonists with a specific signaling response [67]. Based on this information, this study succeeded in predicting additional ligands with a tailored signaling profile. Such a strategy has been also successfully applied to ligands targeting the dopamine D_2_ [68], M_2_ [69], and AT_1_ [70] receptors. 

Moreover, MD studies can also capture downstream events related to signaling bias. In this respect, novel mechanistic insights revealed the connection between ligand binding, conserved micro-switches, and arrestin bias in serotonin receptors [71]. In particular, simulations showed that interactions of the ligand with the binding pocket determine the rotational freedom of TM6 which, in turn, impacts the conformation of the highly conserved P-I-F motif. Consequently, a hydrophobic connector region between the P-I-F motif and the ionic lock seems to contribute to the formation of a water channel that determines the degree of receptor opening (disrupted ionic lock). This conditions G protein coupling and, thus, whether signaling is biased towards arrestin or not. This work highlights the capacity of MD to shed light on features that cannot be extracted from static structures. Another relevant example was an extensive study developed by Kapoor et al. aiming to explain the basis of functional selectivity in the µ-opioid receptor [72]. Among other findings, the study identified distinct conformational rearrangements in the receptor bound to a balanced or a G protein-biased agonist. They also highlighted differences in the allosteric communication, with a more pronounced transfer of information triggered by the G protein-biased agonist. Finally, Nivedha et al. developed a computational method to predict ligand bias in GPCRs ahead of experiments [73]. For that, they used MD simulation to calculate the mechanism of allosteric communication from the extracellular region to the intracellular transducer coupling region. Additionally, they were able to identify functional hotspot residues that potentiate the ligand-mediated bias, which can greatly aid in the design of biased ligands for GPCRs.

#### 2.2.3. Allosteric Ligand Binding

When studying ligand binding, traditional efforts have focused on targeting the orthosteric binding site of GPCRs. The orthosteric binding site of many GPCR subtypes is highly conserved. As a consequence, orthosteric ligands often target several receptors simultaneously, leading to off-target side effects. This leads to one important challenge of GPCR drug discovery, which is achieving selectivity, the ability of ligands to specifically target one receptor subtype over another.

Contrarily to orthosteric ligands, allosteric ligands bind to sites topographically distinct from the orthosteric binding site. Such allosteric binding sites are much more variable in terms of the sequence, which gives allosteric ligands the potential to achieve greater selectivity at GPCR subtypes [23,74]. Allosteric ligands modulate the effect of the orthosteric ligand on the target, which provides a strategy to fine-tune cellular responses triggered by the orthosteric ligand. These characteristics of allosteric ligands have invited a growing interest in designing drugs that target allosteric pockets of GPCRs [75,76]. 

However, targeting allosteric sites comes with some challenges. Allosteric binding sites are not evident from crystal structures. Moreover, the molecular mechanisms by which these modulators affect GPCR signaling depend on dynamical properties that are not evident from static structures. This makes computational methods such as MD simulations a valuable approach to detect hidden allosteric binding sites and determine the mechanistic basis of allosteric regulation [77]. MD-based studies have been especially helpful for the identification of allosteric mechanisms in muscarinic receptors, which are usually paradigmatic for all GPCRs [78,79,80]. One case is the work from Dror et al. in which they provided a structural basis of allosteric ligand binding and described mechanisms of cooperativity between the allosteric and the orthosteric ligand [80]. In another study, Chan et al. applied long-timescale MD simulations to show that acetylcholine, the endogenous ligand, can go from the orthosteric binding site into a deeper allosteric binding site [81].

### 2.3. Revealing the Dynamic Behavior of Water Molecules and Ions

Comparative analysis of available crystal structures pointed to the relevance of waters for receptor dynamics and function [82]. The unique ability of MD to monitor diffusion and binding events of all water molecules in a system enabled the further elaboration of this idea. Simulations of the opioid receptors revealed that GPCR activation correlates with the entrance of waters from the extracellular side [83,84]. In line with this finding, further studies demonstrated that activation of the A_2A_R is linked with the formation of continuous water channels [85,86]. Detailed investigation of the simulation frames revealed that the formation of this channel is mediated by rearrangements of conserved residues W6.48 and Y7.53, the latter of which forms the NPXXY motif.

Importantly, water molecules also have a strong impact on ligand binding and unbinding events, which can be investigated in detail with MD simulations. It is well established that water has a role in ligand-receptor dissociation. For example, Schmidtke et al. showed that shielding ligand-receptor hydrogen bonds from water can contribute to long ligand residence time [87]. Interestingly, Magarkar et al. recently found, based on MD simulations, that shielding of water from intra-protein interactions, not directly involved in ligand−receptor interactions, is also a relevant factor in ligand binding kinetics, as such interactions confer the rigidity of the binding site [88]. This opened new opportunities for the optimization of the residence time during drug development pipelines.

Similarly, MD simulations helped to shed light on the role of ions for GPCR function. Sodium ions are known to be important allosteric modulators of GPCRs, but the mechanism of this modulation is still not well understood [89]. Using MD simulations, Selent et al. provided structural details on the binding of sodium ions in the D_2_ receptor and proposed the molecular mechanism of the allosteric sodium-induced modulation [90]. Several studies have been dedicated to revealing atomistic insights into allosteric sodium ion binding to other class A receptors [91,92]. For example, Selvam et al. elucidated the sodium binding mechanism of 18 GPCRs based on hundreds-of-microsecond long simulations [93]. Their analysis of the kinetics of sodium binding to the allosteric site revealed key residues that act as major barriers for sodium diffusion. Also, they reported that sodium ions can bind to GPCRs from the intracellular side when the allosteric site is inaccessible from the extracellular side. Furthermore, Vickery et al., based on MD simulations and free energy calculations, suggested that the opening of the conserved hydrated channel in the active M_2_ muscarinic receptor allows the exchange of a sodium ion from its extracellular binding pocket to the cytoplasm. This exchange of sodium could be a key step in class A GPCR activation [94]. Beyond allosteric ion effects, a recent study has also proposed that sodium ions can stabilize ligand binding in the orthosteric site and by this enhance receptor signaling in the D_2_ receptor [95]. 

### 2.4. Impact of Natural Genetic Variants

Another factor that impacts GPCR functionality is genetic variants. A huge number of natural genetic variants are observed in GPCRs, as listed in dbSNP [96] and GPCRdb [4]. To name a few, missense variants in rhodopsin are responsible for retinitis pigmentosa due to an alteration in receptor folding and cellular trafficking [97], and missense variants in the C-C chemokine receptor 6 exhibit loss-of-function effect by decreasing G-protein signaling [98].

Understanding the impact of natural variants on GPCRs is critical, as variants can be responsible for disease susceptibility, as well as distinct responses to treatments [99]. Such functional differences can be caused by alterations in dynamic processes of ligand binding pathways, ligand binding interactions, constitutive receptor activity, or recognition of intracellular effector proteins (e.g., G protein binding). Hence, MD simulations are a promising approach to elucidate the molecular mechanisms that explain functional differences between wild type and variant GPCRs, providing genotypic-phenotypic correlations [100,101,102,103]. MD simulations were used, for example, to determine the molecular basis of the effect of a commonly found variant: the Arg16Gly variant of the β_2_AR. This variant has been linked to a differential response to albuterol, a β_2_AR agonist frequently used in the treatment of asthma. Results revealed that the Arg variant increased the dynamics of the N-terminal region, where this polymorphism is located. This change in dynamics leads to long-range effects at the ligand binding site, altering ligand binding-site accessibility, which is higher in the Gly variant [102]. Similar results were recently found for the Gln27Glu variant of the same receptor, which perturbs the network of electrostatic interactions that connects the N-terminal region with the binding site, altering drug response [103].

### 2.5. Complementing Experimental Maps

A critical step in X-ray crystallography or cryo-EM of GPCRs is fitting the receptor model to the experimental density map. After the fitting procedure, certain density areas often remain unmatched, a piece of information that can be extracted from the so-called difference maps (fo-fc). The discrepancy between model and experimental density map may arise from the existence of different rotameric states or the binding of water molecules and ions. Thanks to MD it is possible to complement static structures with this information by monitoring the dynamics of sidechain rotations and the diffusion/binding of solvent molecules that can justify unmatched density areas. Moreover, MD allows investigating highly flexible regions that explain low-resolution areas in density maps.

## 3. Application of MD in Drug Discovery

The drug discovery process implies an immense cost, high risk, and a long time to move from the bench to the market [104]. Computer-aided drug design has the potential to de-risk and accelerate this process [74], and thus it has become an attractive approach for drug discovery targeting GPCRs [105]. Static structures have proven highly effective at aiding drug design [106]. However, due to the high flexibility of GPCRs, especially in druggable regions such as allosteric sites, the full potential of structure-based drug design requires a deeper understanding of GPCR dynamics [80,107].

One of the most widely used structure-based drug design strategies is virtual screening, where libraries of small molecules are screened to identify those structures which most likely bind to the target. Virtual screening is traditionally based on docking the ligands to a static structure of the target protein. This approach has been very successful for the discovery of new ligands. Yet, docking does not consider the flexibility of the binding pocket, thus leading to the identification of only a subset of binders, namely those similar to the crystallized ligand [14]. Using MD to account for the dynamic behavior of the binding pocket generally increases the diversity of ligands identified [55,108]. Moreover, it allows exploring rare conformations that can help define drugs with higher specificity for the receptor [109]. Overall, MD-based methods are more resource-consuming compared with traditional docking, but a higher accuracy can be reached. For example, an interesting approach to include dynamic information in virtual screening protocols is the characterization of the binding site using MD to construct ensembles with structural diversity, where the ligand candidates are docked [110,111,112].

MD can also provide valuable information to guide lead optimization, where the ligand is modified to improve properties, such as potency, selectivity, or pharmacokinetic parameters. Dynamic information can be used to identify the key interactions that the lead ligand establishes with the binding pocket, as well as rearrangements of the binding pocket induced by the ligand [113]. Simulations can further help test and refine potential ligand poses, or even reveal unknown binding sites [77,114]. They are also valuable to improve selectivity, as they can be used to identify differences in the dynamics of binding pockets of closely related receptor subtypes [113].

Moreover, simulation-based methods were found to provide substantially more accurate estimates of ligand binding affinities (free energies) compared to other computational approaches [115]. For now, it is not possible to sample enough unbinding events to determine rates or affinities by unbiased MD. However, it is possible to combine MD with specialized free-energy techniques to enhance sampling for this purpose. This is the case of the free energy perturbation method, which can be used to evaluate and compare the relative affinity of several compounds, such as derivatives of a particular ligand, on a target receptor. This was shown to be particularly useful, for example, for fragment optimization [116]. Similarly, this technique can be used to characterize and compare the effect of single-point mutations of residues in the binding pocket on the binding affinity of a ligand, which helps to determine its binding mode [117]. Another extended approach is metadynamics simulations. Provasi et al. pioneered the use of metadynamics [13] to study ligand binding to GPCRs [118] and have successfully applied this enhanced MD algorithm to predict the binding pose of several orthosteric and allosteric ligands in opioid receptors [119,120]. Still, automatizing metadynamics protocols in drug discovery workflows is challenging, since they usually require specific testing and optimization, mainly to select adequate collective variables [121]. However, efforts are being made to generate accurate and inexpensive metadynamics protocols that can be applied to a broad range of different GPCRs and ligands. This is the case of Saleh et al., who proposed a generally applicable metadynamics protocol that uses a single, optimal CV to accurately and efficiently explore the entire ligand binding path and predict binding mechanisms and affinities [122]. 

Another important application of simulation techniques for lead optimization is the optimization of drug binding and unbinding kinetics, which plays a critical role in drug efficacy, selectivity, and safety [58,59,60,61,62,63,65,123]. In fact, the ligand unbinding kinetics (the inverse of its residence time on the protein) is sometimes better correlated with drug efficiency than binding affinities [124]. Successful examples like tiotropium demonstrate the potential of kinetic optimization. Tiotropium is a well-known M_3_ muscarinic receptor antagonist used as treatment for chronic obstructive pulmonary disease. Its very slow dissociation rate from the M_3_ receptor is postulated to be the key to its superior pharmacological profile. Interestingly, while tiotropium has a similar affinity for the M_2_ and M_3_ receptors, it shows kinetic subtype selectivity towards the M_3_ [125]. Based on MD simulations, this selectivity was found to be caused by differences in the electrostatics and flexibility of the extracellular surface [126].

To further decipher the molecular basis of binding and unbinding kinetics, MD simulations can be used to obtain the whole binding pathway of the ligand, identify metastable binding sites and detect the energetic barriers that govern drug binding and unbinding kinetics [127]. Ligand dissociation time scales are often much longer than those accessible by unbiased MD, even when specialized hardware is used. Thus, enhanced sampling algorithms such as metadynamics are commonly employed. To increase the applicability of these techniques in drug discovery, several variations of conventional metadynamics protocols are created, for example by combining metadynamics with adiabatic-bias MD [128,129].

When designing a GPCR-targeted drug, one aims to achieve a particular signaling profile. In other words, the drug needs to be able to stabilize certain conformational states of the receptor. This is a complex process that requires an understanding of how subtle changes in the binding pocket lead to different conformations of the intracellular coupling interface and, in turn, different signaling profiles. Achieving the desired signaling profile is especially challenging in the case of biased ligands. The successful design of a biased ligand requires knowledge of the conformations associated with G protein signaling and arrestin signaling. As discussed in previous sections, MD simulations are able to provide detailed information on binding pocket dynamics and allow us to compare the receptor-ligand interactions that occur in different conformational states of the receptor and in complex with different types of ligands (e.g., unbiased agonist, biased agonist, inverse agonist, or antagonists) [14]. This opens the road for a more tailored and fine-tuned drug development.

## 4. Workflow for MD Simulation

**Figure 3:**
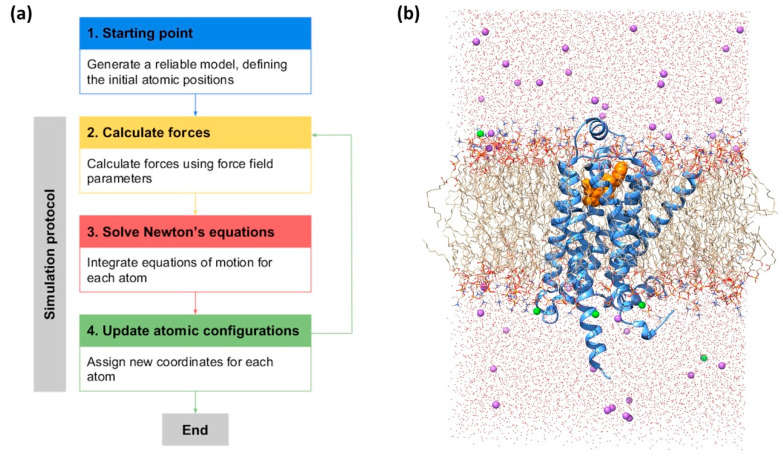
(**a**) Flowchart summarizing the stages of a MD simulation. (**b**) Example of a GPCR molecular system, including the β-2 adrenergic receptor (β2AR, blue) with a full agonist in the binding site (orange) in a 1-palmitoyl-2-oleoyl-sn-glycero-3-phosphocholine (POPC) membrane (tails in light brown, heads colored by heteroatom). The system is solvated with water (red) and ionized with sodium (green) and chloride (purple) ions.

The procedure for conducting MD simulations can be divided into four stages (Figure 3a). In the first stage (stage 1), we create the initial coordinates for our simulation system (Figure 3b). This generally involves the curation of experimentally solved receptor structures (e.g., modeling of missing residues/loops, reverting thermo-stabilizing mutations to the wild-type, etc.) or the application of homology modeling. The obtained GPCR model is then embedded into a specific membrane, solvated, and ionized to a physiological concentration. In this initial stage, one should carefully consider factors such as atomic resolution (atomic scale versus coarse-grained), absence or presence of post-translational modifications (palmitoylation, phosphorylation, glycosylation), and the composition of the membrane environment. 

Once the starting structure is obtained, we proceed to simulate the atomic motions of the system. For that, the forces that act on each atom in the system are calculated (stage 2). This is possible thanks to the so-called force fields, a set of empirical potential energy functions that include all parameters needed to solve both bonded and non-bonded atomic interactions [130,131]. Based on the obtained forces, the atomic positions at the following timestep are predicted by solving the classical (i.e., Newtonian) equations of motion (stage 3). Then, the positions of the atoms are updated accordingly (stage 4). From here, we start an iterative cycle by re-calculating again the forces that act on each atom in the new conformation of the system (stage 2), solving Newton’s equations (stage 3), and updating the atomic positions (stage 4). The time length between these iterations, known as the simulation timestep, should be shorter than the fastest process in the system (typically the vibrations of bonds between heavy atoms, as we commonly constrain hydrogen atoms) and usually is around 2 fs.

The timescale of the biological process we are interested in defines the number of iteration steps needed to complete the simulation, which can easily be higher than millions. Knowing when to stop a MD simulation is not trivial, but one has to ensure that the simulation has efficiently sampled the conformational space of the biological process studied.

## 5. MD Analysis—Extracting Data from the Simulations

### 5.1. Principles of MD Analysis

Due to the extensive amount of information generated by MD simulations, specific computational tools have become mandatory for their proper analysis. Some of the most popular tools include python modules such as MDAnalysis [12,13] and MDtraj [132], which allow the automatization of analysis pipelines using scripts. The visualization and modeling software Virtual Molecular Dynamics (VMD) [133] also provides a range of analysis tools that can be expanded even further by using plugins. In addition, simulation software like GROMACS [134] and CHARMM [135] include their own build-in sets of analysis tools. Even more, there exist online repositories such as Plumed-nest [136], specifically developed to store scripts used for generating and analyzing MD simulations. Despite the diversity in available tools, certain parameters are frequently analyzed, as they provide relevant information about the simulation. 

**Figure 4:**
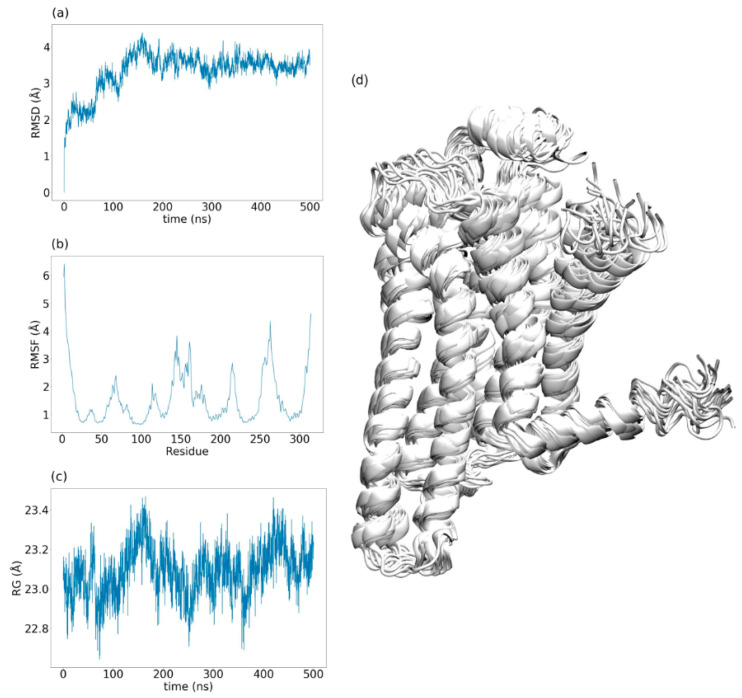
Example of different parameters analyzed in a 500 ns-long MD simulation of the A_2A_ receptor (A_2A_R). (**a**) Root mean square deviation (RMSD) profile taking as reference the first frame of the simulation, which is superimposed to the rest of the frames. RMSD values (i.e., structural differences with respect to the reference frame) increase over the simulation time until the system reaches a stable conformation after 100 ns. (**b**) Root mean square fluctuation (RMSF) profile displaying the values of all the alpha carbons in the protein. Higher RMSF values correspond to flexible loops, while lower ones belong to transmembrane helices, where residues are stabilized by the secondary structure. (**c**) Radius of gyration (RG) profile where the RG fluctuates around the same value during the simulation, indicating that the system does not suffer any big change in compactness. (**d**) Superimposition of 25 representative frames of the simulated receptor. The relative mobility of loop regions contrasts with the rigidness of the transmembrane helices.

One of the most important parameters is the root mean square deviation (RMSD), which allows a quantitative evaluation of the structural changes that occur during a simulation. It is based on the distances between the atoms of the protein at a certain frame and the same atoms at a superimposed reference frame (Figure 4d). The RMSD is obtained with the following equation:

$$\mathrm{RMSD}=\;\sqrt{\frac{1}{n}\overset{n}{\underset{i=1}{\sum }}\|x_{i}\left(t_{j}\right)-\;x_{i}{\left(t_{0})\right\|}^{2}}$$

where $x_{i}\left(t_{j}\right)$ represents the coordinates of atom *i* at frame *j,* $x_{i}\left(t_{0}\right)$ represents the position of the same atom *i* at the reference frame, and *n* the number of atoms in the system.

RMSD profiles (i.e., RMSD over time) are routinely used to assess the stability of the simulated protein and detect transitions between different conformations (Figure 4a). It is also useful to compare the dynamic behavior of the receptor under different conditions, as done by Ozcan et al. to determine the effect of the intracellular loop 3 in human β_2_AR [137].

Another widely used parameter is the root mean square fluctuation (RMSF), which describes the relative mobility of an atom or residue in the simulation. The RSMF is based on the mean square of the residue or atom position in each frame, which can be obtained using the following equation:

$$\mathrm{RMSF}=\;\sqrt{\frac{1}{T}\overset{T}{\underset{j=1}{\sum }}{\left(x_{i}\left(t_{j}\right)-\;\bar{x_{i}}\right)}^{2}}$$

where $x_{i}\left(t_{j}\right)$ represents the coordinates of atom *i* at frame *j*, $\bar{x_{i}}$ the average position of atom *i* in the simulation and *T* the total number of frames in the simulation.

RMSF profiles (i.e., RMSF as a function of atoms/residues) are often employed to describe and compare the relative mobility of specific regions of the receptor (Figure 4b). For example, Semack et al. were able to detect specific flexibility profiles for the β_2_AR and the vasopressin receptor 1A when bound to different sets of peptides derived from the C-terminus of the G alpha subunit [138].

Furthermore, the radius of gyration (RG) is a valuable parameter to describe the overall compactness of the protein. Specifically, the RG is defined as the mean square of the distance between each protein atom and the center of mass of the protein:

$$\mathrm{RG}=\;\sqrt{\frac{1}{n}\overset{n}{\underset{i=1}{\sum }}{\left(r_{i}-\;r_{cm}\right)}^{2}}$$

where $r_{i}-\;r_{cm}$ represents the distance between atom *i* and the center of mass of the molecule and *n* the total number of atoms in the system.

RG profiles (i.e., RG over time) can be used to assess the evolution of the protein compactness during a simulation (Figure 4c), as done by Davoudmanesh and Mosaabadi to study the effects of homocysteinylation of the neuropeptide substance P on its binding with the NK1 receptor [139].

In order to obtain a detailed view of the molecular mechanisms that drive general receptor properties such as protein stability (RMSD), conformational flexibility (RMSF), and compactness (RG), one needs to analyze the intramolecular interactions. In this respect, non-covalent interactions between residues play an important role. Also, non-covalent interactions are critical for ligand recognition. In MD simulations, these interactions and their stability can be predicted based on atom distances and angles. Using this methodology, Dror et al. were able to discern the importance of an ionic lock interaction for the conformation change produced during the activation of β_2_ARs [140].

**Figure 5:**
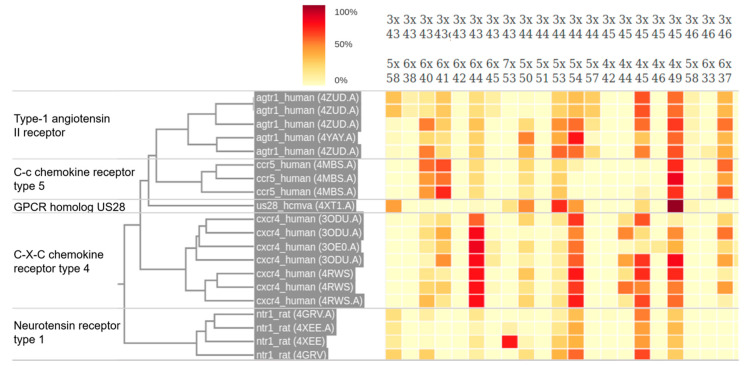
Pattern of total interaction frequency of several MD simulations of GPCRs, extracted from the GPCRmd Receptor Meta-analysis tool (https://submission.gpcrmd.org/contmaps/) of the GPCRmd server [53]. Columns represent interacting residue pairs according to Ballesteros-Weinstein residue numbering [142], whereas rows represent different simulations. The color of each cell shows the frequency in which any type of non-covalent interaction occurs during the simulation. Results are clustered based on the interaction frequencies of the simulations. This clustering is able to separate simulations according to the receptor subtype, showing that different receptor subtypes present differentiated interaction patterns.

Most of the aforementioned MD analysis tools (e.g., MDAnalysis, GROMACS, VMD) focus on hydrogen bond interactions, as this is one of the most abundant and structurally important interaction types in proteins. However, there are many other interaction types that should not be neglected, including van der Waals, salt bridges, π-cation, and π-stacking interactions. To analyze them, more specialized tools have been developed, such as the python module GetContacts [141]. A good example of the capabilities of this module can be found in the Receptor Meta-analysis web tool (https://submission.gpcrmd.org/contmaps/) included in the GPCRmd platform [53]. This tool analyzes and compares different types of non-covalent interactions obtained from a large GPCR simulation dataset using GetContacts scripts, and displays them into a series of interactive plots (Figure 5). This allows extracting conclusions about the interaction pattern of different GPCRs.

### 5.2. Analysis of the Allosteric Communication

Allostery is a property of a protein by which perturbations that take place in one part of its structure are transmitted to distant parts of it. GPCRs are an excellent example of allosteric proteins. As described in Section 2.1, the binding of a ligand in a GPCR causes local structural changes in the binding pocket, which are transmitted across the receptor leading to a global conformational change and, in turn, a specific signaling response. This is mediated by a complex allosteric network in which multiple nodes (i.e., residues) transmit a specific perturbation through the whole protein. Not only GPCRs present this phenomenon. Multiple studies have explored allostery in many other proteins like thrombin and PDZ domains among others [143,144].

An accurate model to capture protein allostery is an important focus of current research efforts. For instance, knowing how a drug candidate affects allosteric communication would be of great help to fine-tune its potency and efficacy in drug development programs. Moreover, protein engineering could be considerably improved if we were able to access the repercussion of a mutation in the protein structure and, thus, its functional outcome.

MD simulation has been used in many studies as a tool to analyze protein allostery. However, the way in which researchers look at this data is heterogeneous. Numerous studies rely on the comparison of the structural and dynamic behavior between two or more conditions. Others focus on analyzing the transition between two conformational states. In this case, the role of metadynamics is crucial, given that some of these transitions happen in time scales not accessible for classical (non-biased) simulations [145]. Another approach is to focus on changes in the conformational space, which is commonly studied using principal components analysis [146]. Others base their studies on the correlations in the movement of residues [147]. For this, the use of information theory-based methodologies is the most common approach to measure dependence between residues or groups of residues [148]. Finally, some researchers pay more attention to variables influenced by the chemical context of the residues, such as the contacts with other residues [149].

In many cases, some of these relationships are used to build networks. In these networks, residues are represented as nodes, while edges represent the level of coupling between the residues. Then, centrality and community analysis can be applied to the network to find the residues that contribute the most to communication inside the protein [147].

Some of the most influential works in the field combine several of the approaches mentioned. For example, Dror et al. studied the conformational correlation of β_2_AR subdomains in different activation states to propose an activation mechanism of this receptor [28]. Also, Miao et al. analyzed metadynamics simulations of the M_2_ muscarinic receptor using a network representation of the residue cross-correlation [150]. This analysis allowed them to characterize some aspects of the receptor activation. Finally, Bhattacharya and Vaidehi investigated network representations of the inter-residue dihedral correlation of the β_2_AR [151]. The resulting model describing allosteric communication was able to identify allosteric pockets and identify residues that affected function upon mutation.

Overall, this field has a great potential for understanding GPCR pharmacology but is still challenging and requires the development of more robust protocols. This robustness might be achieved by integrating the different methodologies that are being used into a more complete analysis.

## 6. Current Challenges

The capabilities of MD simulations have broadened substantially thanks to the technological advances of the last decades. However, there are still some relevant drawbacks that limit the usability of this technique and must be taken into account.

As described in Section 2.1, the forces of a MD simulation are calculated based on a force field, which consists of a set of empirical potential energy functions. Force fields are based on quantum mechanical calculations and experimental measurements, and include some approximations. As such, force fields are imperfect. Studies comparing simulation results with experimental data indicate that force fields have improved significantly over the past decade [152], but more remains to be done to achieve increased accuracy. Another limitation of classical MD is that it is not possible to form or break covalent bonds during the simulation. As a consequence, protonation states of titratable amino acid residues are fixed, as well as disulfide bonds. Thus, they have to be set carefully at the beginning of the simulation [113].

An important challenge that needs to be taken into account is the simulation timescale. The simulation timestep, which is the time length between evaluations of the potential, needs to be small enough to capture the fastest movements in the simulation system. This typically limits the timestep to around 2 fs. Many relevant molecular events, however, take part in the microsecond to millisecond scale, or even longer. This implies the calculation of a vast number of timesteps, each of which involves the calculation of millions of interatomic interactions. As a consequence, reaching long timescales can be challenging for classical MD. Furthermore, the issue with long-timescale events is that they imply the transition between free energy states that are separated by high-energy barriers. In this situation, classical MD simulations tend to get trapped in one of these local minimum-energy states for a long time, which restrains the sampling process. In turn, this leads to a poor characterization of the protein’s dynamic behavior [153]. A useful strategy to tackle the sampling problem is the application of enhanced sampling techniques. Enhanced sampling simulations, including replica-exchange MD, metadynamics, and simulated annealing, are able to efficiently overcome energetic barriers and access additional conformational states by including an external bias [22,154]. Simplified models like coarse-graining can also extend accessible timescales by orders of magnitude, as they are less expensive computationally [155]. Nevertheless, the problem of achieving relevant simulation timescales with classical, all-atom MD seems to be within reach of being solved [156]. In recent years, there has been a dramatic increase in achieved timescales. This tendency is expected to continue, thanks to the advances in algorithms [8,9,157,158], software [159,160,161,162], and hardware [163,164] that we are experiencing. In fact, it is expected that all-atom, classical MD simulations will be able to reach the second timescale within the next five years [165,166,167].

Parallel to the limitation of longer timescales accessible to simulations, there is a limitation in the size of the systems that can be studied. As the system size increases, so does the computational power needed to carry out the simulation. In general, the required computational power increases with the square of the number of atoms involved. Moreover, as molecular systems become bigger, the biologically relevant timescales tend to increase too [165]. Overall, this challenges the study of GPCRs in complex with G protein or arrestin. Enhanced sampling techniques are a promising approach for the study of such systems. However, selecting predefined collective variables for the simulation of protein-protein interactions is a difficult task, as such processes often involve large-scale translations and rotations of the binding partners, as well as complex conformational changes. Thus, methods that do not require predefined collective variables, such as Gaussian accelerated MD, are especially convenient. In fact, recently Miao et al. successfully applied Gaussian accelerated MD to simulate the intracellular association between the M_2_ receptor and a G-protein mimetic nanobody [168]. Their simulations revealed important insights into the binding mechanism, despite the fact that the calculated free energies were not converged. Future developments will be needed to achieve converged simulations of such complex systems.

Given this fast evolution of the capabilities of MD simulation, this technique is gaining more and more relevance. In view of this, it is becoming increasingly necessary to define standards and best practices to ensure a reproducible research output [169]. Many challenges remain in order to effectively reach this goal. One issue is the creation of workflows for simulation production and analysis. The file formats and force fields supported by different programs are often incompatible. This limits the combination of software packages that can be used together in a workflow and restricts the choice of algorithms and force fields based on software compatibility rather than scientific-based reasons. Luckily, this can be solved with the development and usage of software that converts molecular information between the different file formats. Still, this does not solve the problem that different programs, or program versions, may implement force fields and features, such as thermostats and integrators, in different ways. Thus, the results of a workflow will be influenced by the combination of programs used [170]. Because of this, it is always important to disclose the version and name of all programs used. In fact, detailed documentation of the entire workflow should always be provided when publishing a simulation. The level of detail in documenting the workflows needs to be enough to ensure the reproducibility of the obtained results. Finally, another challenge that needs to be overcome to achieve reproducibility is data sharing. Data sharing is still not widely adopted in the field of MD simulation, partly because of the technical difficulties derived from the increasing size of the generated trajectories. More efforts should be done to define best practices and guidelines for simulation data sharing [171]. Luckily, many researchers work to promote it [172], and different initiatives are addressing this issue. Several software packages [173,174,175,176] have been developed to share trajectories by providing online interactive visualization based on the advantages of the WebGL API. Moreover, several community-driven projects provide specialized platforms for deposition and analysis of MD simulations [53,136,177,178,179,180,181]. In the case of GPCRs, GPCRmd [53] is an online resource specialized in the deposition and analysis of GPCR MD simulations.

## 7. Conclusions and Perspectives

MD simulations are a potent computational technique capable of generating high-resolution simulations of the structural motions of a molecular system. They can either capture atomic-level motions within a specific conformational state or structural transitions between different conformational populations, bringing within reach information that is difficult, or even impossible, to obtain by other methods [14]. This makes MD simulation a promising technique for the study of GPCRs, whose functionality is highly determined by their ability to transition between conformations. In fact, MD simulations have proven their usefulness for the study of important biological processes in GPCRs such as ligand binding, allostery, activation, natural genetic variation, and addition of post-translational modifications, among others. Since GPCRs are drug targets of striking importance in the pharmaceutical industry [182], all this information generated by MD simulations has the potential to accelerate the discovery of new and improved drugs targeting these proteins.

In order for MD simulation to reach its full potential, some difficulties need to be overcome. Fortunately, we are in an era of rapid technological development, which creates great prospects for the advancement of this field in the following years. Computational power is expected to continue increasing following Moore’s law, which describes how the performance of integrated circuits has been increasing exponentially over the past half-century [183]. This would imply a reduction in computational costs. At the same time, we expect methodological advances in MD algorithms, including improvements in the fine-tuning of energy calculations, parallelization, GPU exploitation, and algorithmic methods to increase the sampling of conformational space. Overall, this would cause an increase in the timescales available to simulations. Several authors propose that we may even reach the second timescale within the next five years [165,166,167], bridging the gap between the timescales of biological processes observed in vivo and those accessible in silico. Parallel to timescales would come a growth in the size of the systems that can be studied [165]. This, together with an ever-growing accuracy in the force fields, will grant us the opportunity to extend the application of MD simulations to the study of processes that were previously difficult to capture. This may open the door to significantly advance in the study of macromolecule-macromolecule interactions [184], including GPCR oligomerization, and coupling to intracellular signaling proteins. While coarse-grained MD has been typically used for this type of study [185,186], it is important to capture the effects of macromolecule-macromolecule interactions on the structural dynamics and cell signaling through more detailed MD simulations [107].

Finally, as simulations become faster, cheaper, and more widely accessible, new opportunities will arise for drug discovery. In the past, most drug discovery programs have disregarded MD analysis because of their computational expenses. With the forthcoming reduction of the computational costs associated with MD simulations, this technique is expected to be more commonly applied in the pharmaceutical industry and, eventually, to be commonly included in drug discovery pipelines [184,187,188].

## Abbreviations

GPCR	G protein-coupled receptor
Cryo-EM	Cryo-electron microscopy
MD	Molecular dynamics
GPU	Graphical processor unit
β_2_AR	β-2 adrenergic receptor
A_2A_R	A_2A_ receptor
TM	Transmembrane helix
MSMs	Markov state models
POPC	1-palmitoyl-2-oleoyl-sn-glycero-3-phosphocholine
VMD	Virtual Molecular Dynamics
RMSD	Root mean square deviation
RMSF	Root mean square fluctuation
RG	Radius of gyration
